# Multifunctional Downhole Drilling Motor Speed Sensor Based on Triboelectric Nanogenerator

**DOI:** 10.3390/mi15111395

**Published:** 2024-11-18

**Authors:** Yanbo Zhang, Shida Su, Lei Zhang, Yulin Gao, Chuan Wu

**Affiliations:** 1 Shenhua Geological Exploration Co., Ltd., Beijing 100089, China; 10018120@ceic.com (Y.Z.); 18910962002@163.com (L.Z.); 2 College of Engineering and Technology, China University of Geosciences (Beijing), Beijing 100083, China; 3 Faculty of Mechanical and Electronic Information, China University of Geosciences (Wuhan), Wuhan 430074, China; wuchuan@cug.edu.cn

**Keywords:** triboelectric nanogenerator, self-powered, rotational speed sensor, rotational direction, rotational angle

## Abstract

The measurement of downhole drilling motor rotational speed is crucial for optimizing drilling operations, improving work efficiency, and preventing equipment failures. However, traditional downhole rotational speed sensors suffer from power supply limitations, which can increase drilling costs. To address this issue, this study presents a novel multifunctional rotational speed sensor based on triboelectric nanogenerator (TENG) technology, enabling the self-powered measurement of rotational speed, direction, and angle. Our experimental results demonstrate that the sensor operates stably within a temperature range of 0 to 150 °C and a humidity range of 0 to 90%. It achieves rotational speed measurement with an accuracy of less than 2.5% error within a range of 0 to 1000 rpm, angular measurement with a resolution of 60 degrees and an error of less than 2% within a range of 0 to 360 degrees, and rotational direction measurement. Furthermore, the sensor exhibits self-powered functionality, achieving a maximum power output of 29.1 μW when the external load is 10 MΩ. Compared to conventional rotational speed sensors, this sensor possesses the unique advantage of integrating the measurement of rotational speed, angle, and direction, while simultaneously harnessing downhole working conditions for self-power generation. These characteristics make it highly suitable for practical downhole environments.

## 1. Introduction

Drilling is a technology that uses specialized equipment to create holes from the earth’s surface into its interior. It is a crucial technological means for the exploration and development of mineral resources [1]. The main components of drilling equipment include the drilling rig, drill pipe, and drill bit. The drill pipe is a thin-walled circular tube of a specific length that rotates under the drive of the drilling rig, using rotational force to drive the drill bit at its end to break rock and achieve downward penetration [2,3]. When the drilling depth is significant, the surface drilling rig’s power may struggle to reach the bottom of the well, necessitating the use of bottom-hole power drilling tools. As drilling progresses, the drilling trajectory may become inclined. Due to the resistance along the trajectory, the downhole tools may not rotate immediately, meaning that the surface rotational speed and the downhole rotational speed are not the same. The drill pipe connects to the bottom-hole power drilling tool to form a drill string. Changes in the rotational speed of the drill string at the bottom reflect the cutting efficiency and rock-breaking performance [4,5]. Drillers can develop appropriate drilling techniques based on speed parameters. Furthermore, in complex geological conditions, the rotational speed data are also crucial for the subsequent evaluation of mineral resources and mining planning. Therefore, accurately measuring the rotational speed of the drill string at the bottom is essential for optimizing drilling operations, improving work efficiency, and preventing equipment failures.

Current commonly used methods for measuring rotational speed in industry include the Doppler method [6], electromagnetic method [7], and optical method [8,9,10]. While these techniques perform well on the surface, they exhibit limitations in real drilling environments, primarily because all sensors require an external power supply. Rotational speed sensors are installed at the bottom of the borehole, and the current power supply method for downhole rotational speed sensors mainly relies on batteries [11,12]. When the battery at the borehole bottom depletes, the entire drilling assembly must be lifted to the surface for battery replacement. This process can take several days when the drilling depth reaches several kilometers, resulting in huge costs and significantly affecting drilling efficiency. Therefore, if downhole rotational speed sensors could possess self-power generation capabilities, this would effectively reduce reliance on external power sources, thus enhancing drilling efficiency and lowering drilling costs, making them more suitable for actual drilling conditions.

The emergence of triboelectric nanogenerator (TENG) technology has brought hope for addressing the power supply challenges faced by sensors. Friction refers to the force that hinders the movement between the contact surfaces when two objects in contact are in relative motion or have a tendency to move relative to each other. Proposed by Z.L. Wang [13], TENGs are based on the principles of triboelectricity and electrostatic induction from nanomaterials and have found extensive applications in the fields of energy generation and sensing. For example, in the energy generation sector, real-time energy harvesting and power generation have been achieved from wind energy [14,15,16], wave energy [17,18], biomass energy [19], rotational energy [20,21,22], vibrational energy [23,24], acoustic energy [25], and raindrop energy [26,27]. In the sensor domain, TENGs have successfully measured various parameters such as angle [28], acceleration [29,30], gesture [31], gas [32,33,34], displacement [35], vibration [36,37], trajectory [38,39], pressure [38], and pulse [40]. Therefore, triboelectric nanogenerators can effectively resolve the power supply issues for downhole rotational speed sensors.

Based on this, the present study develops a self-powered multifunctional speed sensor grounded in the principles of triboelectric nanogenerators. This sensor is capable of simultaneously measuring the rotational speed and angle of the bottom-hole power drilling tool, effectively addressing the dependency of traditional sensors on external power sources. The findings of this research not only contribute technological innovations to while-drilling measurement but also expand the application scope of triboelectric nanogenerators. The results are expected to enhance drilling efficiency and reduce drilling costs.

## 2. Structure and Work Principle

### 2.1. Structure Composition

**Figure 1:**
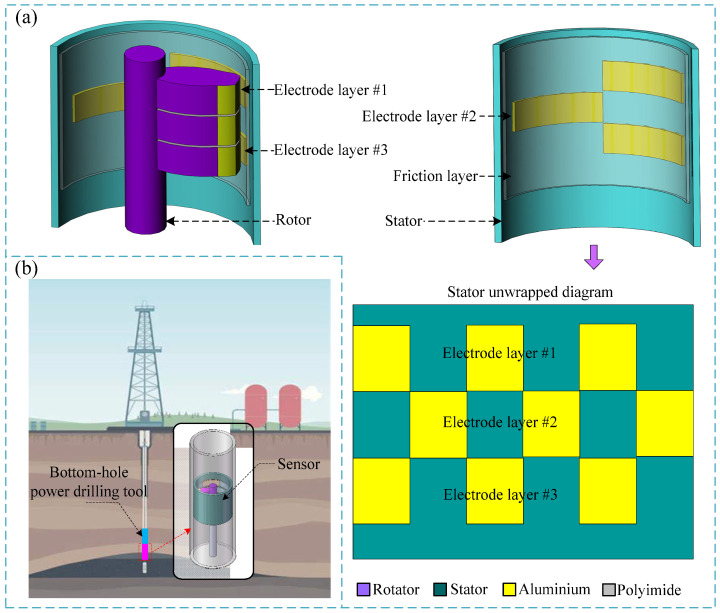
Schematic diagram of the sensor components. (**a**) 3D view of the sensor structure; (**b**) schematic diagram of the sensor installation position.

As shown in Figure 1a,b, the sensor has a tubular structure with an outer diameter of 75 mm, primarily consisting of a rotor and a stator. During actual use, the sensor is installed below the bottom hole power drilling tool, with the rotor and stator of the sensor connected to the rotor and stator of the drilling tool, respectively. The inner wall of the sensor stator is coated with three layers of 0.3 mm thick aluminum (Al) foil as electrode layers, designated as electrode layer #1, electrode layer #2, and electrode layer #3. The surfaces of all electrode layers are covered with a 0.1 mm thick polyimide (PI) film, serving as a friction layer. Although the three electrode layers do not fully cover the inner wall of the stator, the PI film completely encases it. As illustrated in Figure 1a, the electrode layer distribution is as follows: the stator is evenly divided into six segments along the circumferential direction, with each segment occupying a 60-degree angle. An electrode is attached every 60 degrees, and the phase difference between adjacent electrodes is 60 degrees.

### 2.2. Work Principle

The friction between the PI layer on the stator and the friction layer on the rotor generates a triboelectric nanogenerator, with all aluminum (Al) foils on the stator serving as electrode layers. When the rotor rotates, due to the phenomena of triboelectric charging and electrostatic induction, all three Al electrode layers produce periodic electrical signals. Any of these periodic signals can be used to measure the rotational speed. Additionally, because the arrangement of the three Al electrodes is systematically different, the measurement of the rotational angle can be achieved based on the sequence and quantity of the electrical signals generated by the Al electrodes. The specific working principle is as follows.

**Figure 2:**
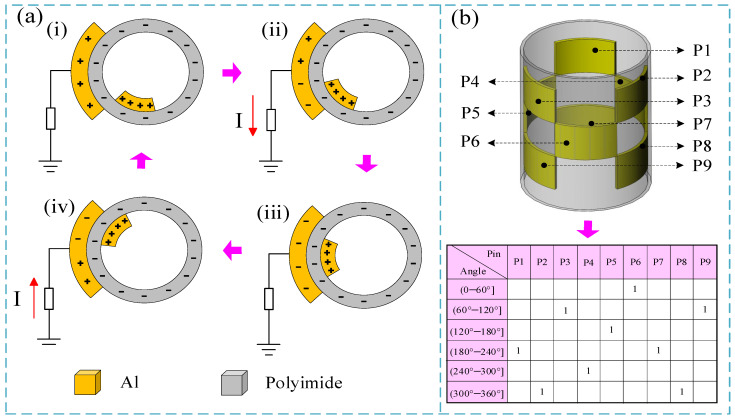
Working principle of the sensor. (**a**) Schematic diagram of the speed measurement principle; (i) Initial state; (ii) Progressive contact condition; (iii) Full contact condition; (iv) Progressive separation state (**b**) schematic diagram of the angle measurement principle.

Figure 2a illustrates the working principle of speed measurement by the sensor. Since the operating principle for generating electrical signals from the three Al electrodes during speed measurement is identical, we will use electrode #3 as an example. As shown in Figure 2a(i), when the rotor rotates, the friction layer generates charge through its interaction with the PI layer. Because aluminum metal is more likely to lose electrons, the friction layer becomes positively charged while the PI layer becomes negatively charged. As the rotor turns, the friction layer gradually aligns with the electrode layer, as depicted in Figure 2a(ii). Due to electrostatic induction, the amount of positive charge on the electrode layer gradually decreases, resulting in a current flowing to the ground. When the rotor reaches a state where the friction layer completely overlaps the electrode layer, as shown in Figure 2a(iii), the positive charge transfer is complete, and the electrode layer becomes negatively charged. As the rotor continues to rotate to the state shown in Figure 2a(iv), the electrode layer and the friction layer start to separate. At this point, the attraction from the positive charge on the stator weakens, causing the negative charge on the electrode layer to gradually decrease until it becomes positive. Simultaneously, a current flows toward the electrode. When the rotor rotates back to the state shown in Figure 2a(i), the electrode layer and the friction layer are completely separated, completing the reverse charge transfer, and the electrode becomes positively charged. This cycle repeats continuously. Thus, within a single rotation cycle, an alternating pulse or voltage pulse signal is generated in the circuit. Consequently, by counting the number of pulse signals within a unit time, the rotational speed can be calculated.

Figure 2b provides a schematic of the working principle for angle measurement by the sensor. All electrodes on the stator are numbered and designated as P1 through P9. As the rotor rotates and makes contact with different positions on the three electrode layers, the corresponding electrodes generate electrical pulse signals upon contact, which are marked as a high level 1. By recording the output level state of the corresponding electrode pins at different angles, we can compile the encoding table shown in Figure 2b. Hence, by utilizing a subsequent microprocessor chip to count the output level states of the different coded pins, discrete angle measurements can be achieved, with a resolution of 60 degrees.

## 3. Experiments and Analysis

### 3.1. Experimental Setup

**Figure 3:**
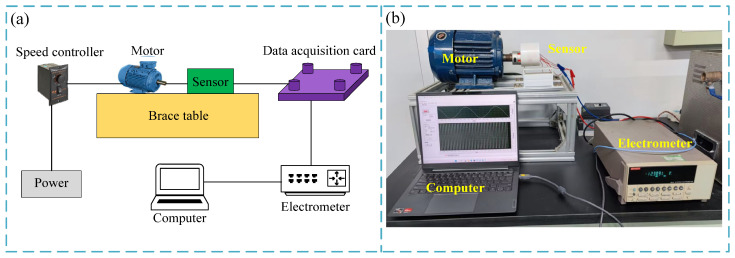
Experimental setup. (**a**) Schematic diagram of the experimental setup. (**b**) Photograph of the experimental setup.

As shown in Figure 3, an indoor simulation device was constructed for the experiment. The motor simulated the rotation of the downhole drill string, and its speed was controlled by a speed controller. The motor output shaft was connected to a sensor. The sensor output signal was processed by a data acquisition card and an electrometer, and then connected to a computer. The computer was equipped with software programmed by LabVIEW, which could realize the real-time display and storage of the sensor output data. During the experiment, the sensor output signal characteristics, speed measurement, angle and direction measurement, power generation characteristics, and environmental adaptability were tested, respectively. The specific experiments are as follows.

### 3.2. Output Signal Characteristic Experiment Results

**Figure 4:**
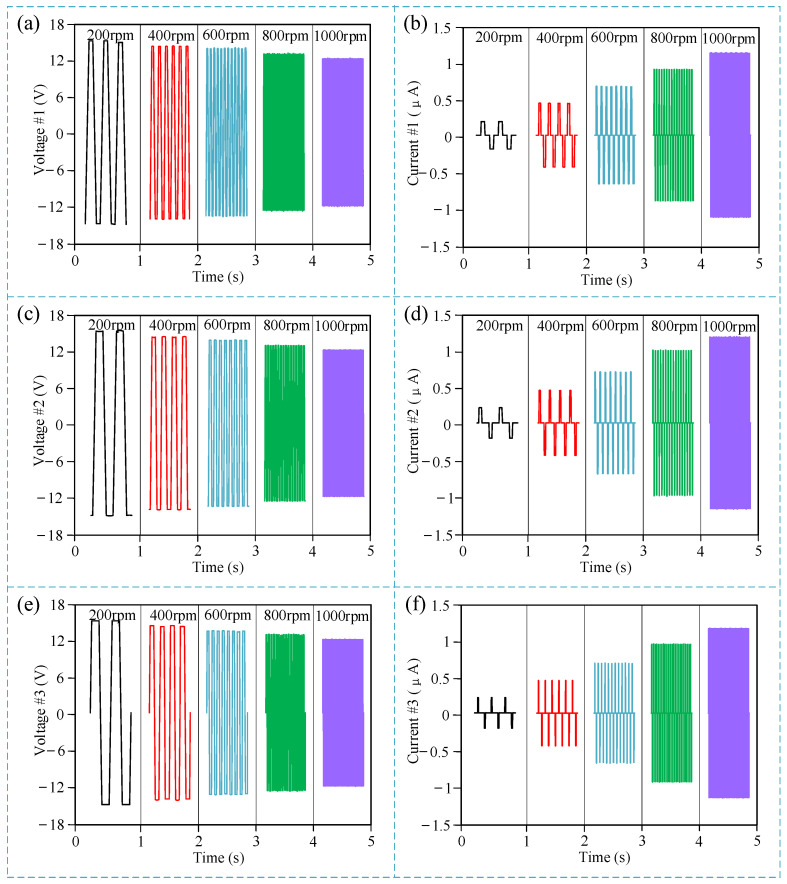
Experiment results of the output signal characteristic. (**a**) Output voltage waveform of electrode #1 at different rotation speeds; (**b**) output current waveform of electrode #1 at different rotation speeds; (**c**) output voltage waveform of electrode #2 at different rotation speeds; (**d**) output current waveform of electrode #2 at different rotation speeds; (**e**) output voltage waveform of electrode #3 at different rotation speeds; (**f**) output current waveform of electrode #3 at different rotation speeds.

The experiment began by testing the output signal waveforms of the three electrodes at different rotation speeds, as shown in Figure 4, the x-axis represents time, and the y-axis represents output voltage and output current, showing the output voltage and current waveforms within 1 s at different rotational speeds. Since the rotation speed range of downhole power drilling tools is less than 1000 rpm, the measurement range of the rotation speed in the laboratory experiment was limited to between 0 and 1000 rpm, which is consistent with actual working requirements. As shown in Figure 4a,c,e, it can be observed that the output voltage of different electrodes of the sensor decreases slightly with increasing rotation speed. The reason is that when the rotation speed increases, the friction between the stator and rotor becomes insufficient [41]. Since the output voltage of a triboelectric nanogenerator is theoretically proportional to the contact area and the contact force, the rotational speed itself does not directly affect the output voltage. However, as the rotational speed increases, the contact between the two frictional surfaces may become insufficient. This results in a reduction in the relative contact area, and insufficient frictional force can cause a decrease in the output voltage. In other words, while the increased speed does not directly affect the voltage, the decreased efficiency of contact at higher speeds leads to lower voltage generation. As shown in Figure 4b,d,f, it can be observed that the output current of different electrodes of the sensor shows a significant increase with increasing rotation speed. This is because, theoretically, the amount of charge generated by each frictional contact remains constant. However, when the rotational speed increases, the accumulated charge per unit of time increases. According to the current calculation formula, as shown in Equation (1), the output current actually increases.

*I = dQ/dt* (1)

Here, *I* is the current, *Q* is the charge, and *t* is the time.

The results shown in Figure 4 reveal that the three electrodes exhibit identical output characteristics. Therefore, for the purpose of rotation speed measurement, any one of the three electrodes can be used as the output signal for the rotation speed sensor. Consequently, subsequent discussions on the rotation speed measurement function will be based on tests conducted using a single electrode. Furthermore, even if an electrode in the sensor is worn or damaged, as long as at least one electrode remains operational, the rotation speed measurement function of the sensor will continue to work normally. This demonstrates the high reliability of the sensor.

### 3.3. Rotation Speed Measurement Experiment Results

**Figure 5:**
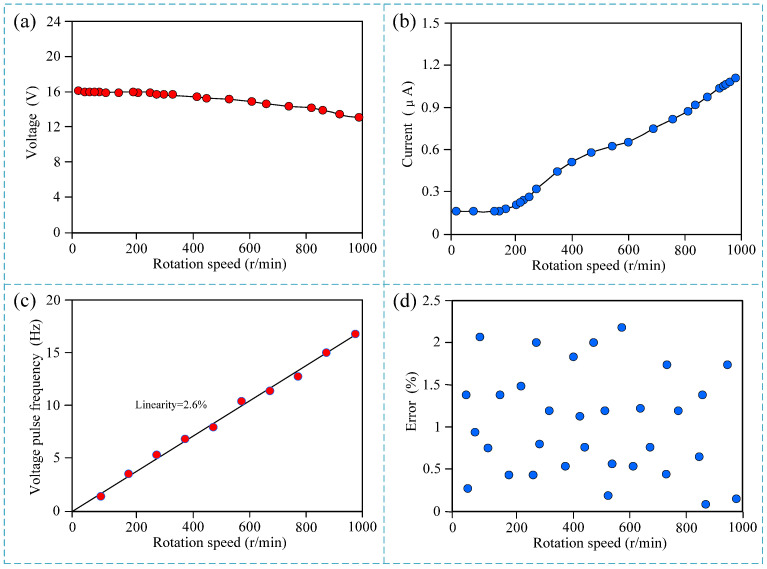
Experiment results of the rotation speed measurement. (**a**) Output voltage at different rotation speeds; (**b**) output current at different rotation speeds; (**c**) output voltage pulse frequency at different rotation speeds; (**d**) scatter plot of measurement errors at different rotation speeds.

The experimental results of the rotation speed measurement are shown in Figure 5. As can be seen from Figure 5a,b, with the increase in rotation speed, the output voltage amplitude of the sensor decreases slightly while the output current amplitude increases significantly. The voltage amplitude varies from 13 V to 16 V, while the current amplitude varies from 0.15 μA to 1.15 μA. The voltage amplitude decreased by 23%, and the current amplitude increased by 667%. Theoretically, both voltage and current signals can be used as output detection signals of the sensor. However, the minimum value of the voltage output signal is 13 V, which is in the order of V, while the minimum value of the current output signal is 0.15 μA, which is in the order of 100 nA. Compared with the voltage value, the current value is relatively weak, and the current value of this order of magnitude is easily affected by noise and introduces large measurement errors. Indeed, both voltage and current signals are typically measured by counting the number of pulses. However, the voltage signal tends to have a larger amplitude, which gives it a stronger ability to resist interference compared to current signals. This is because voltage is often less sensitive to the small fluctuations in the circuit that might affect current, making voltage measurements more reliable in noisy environments. Therefore, the voltage signal is used as the output signal of the sensor in practical applications.

Further calibration was performed on the sensor, and the number of output voltage pulse signals at different rotation speeds was statistically analyzed. The experimental results are shown in Figure 4c. It can be seen that the linearity of the calibration curve is 2.6%. Theoretically, there is an absolute linear relationship between the rotation speed and the output pulse, but the actual situation shows that the linearity of the calibration curve is not 0, which proves that there are measurement errors. Further experiments on the measurement error were conducted, and 1000 groups of experiments were performed in the range of 0 to 1000 rpm, with each group of experiments lasting 5 min. Part of the representative experimental error scatter plots are shown in Figure 4d. It can be seen that the measurement error of the sensor is less than 2.5%, which meets the requirements of practical engineering applications.

### 3.4. Rotation Angle and Direction Measurement Experiment Results

**Figure 6:**
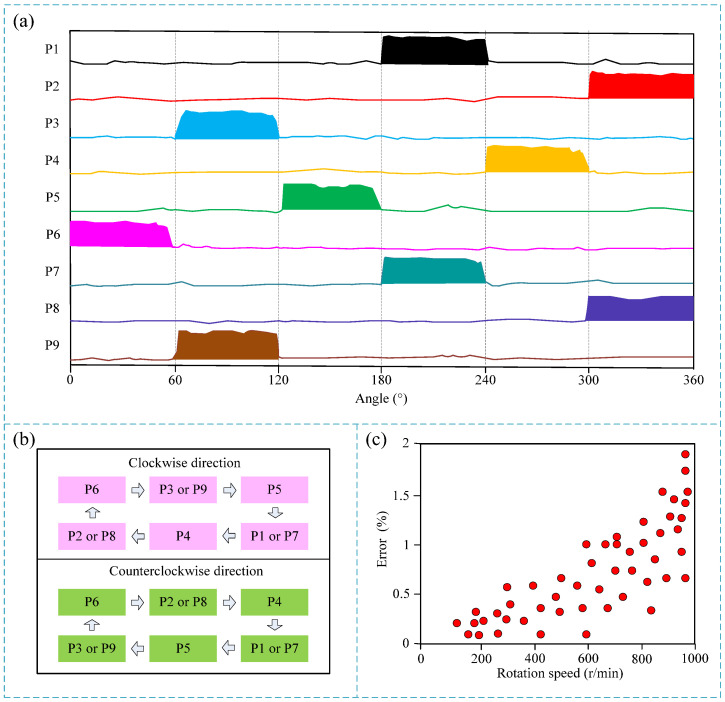
Experimental results of angle and direction measurement. (**a**) Angle measurement experimental results; (**b**) direction measurement principle; (**c**) scatter plot of angle measurement error at different speeds.

Figure 6 shows the experimental results of rotation angle and direction measurement. As the rotor rubs against the electrodes with different position numbers on the stator, corresponding electrical pulse signals are output, thereby achieving rotation angle measurement. As shown in Figure 6a, the curve of the actual experimental results shows that when the rotation angle is between 0 and 60 degrees, the P6 pin outputs a high voltage signal while the other pins are at low voltage. By analogy, the rotation angle can be measured according to the voltage state of the corresponding pins. However, it is important to note that the resolution of the rotation angle measurement is 60 degrees. Further, as shown in the rotation direction measurement principle in Figure 6b, during the clockwise rotation of the sensor rotor, the order of the high voltage outputs is P6 pin, P3 or P9 pin, P5 pin, P1 or P7 pin, P4 pin, and P2 or P8 pin, and so on. Conversely, when the sensor rotor rotates counterclockwise, the order of the high voltage outputs is P6 pin, P2 or P8 pin, P4 pin, P1 or P7 pin, P5 pin, and P3 or P9 pin. Therefore, after connecting the subsequent microprocessor to the sensor, the rotation direction can be measured by the microprocessor judging the order of the pin energization. Furthermore, the angle measurement error was tested. Since the angle measurement principle is essentially realized by friction between electrodes at different positions, the measurement errors for different angle values are the same. However, the speed of rotation affects the signal output effect of the electrode friction. Therefore, the measurement error at different speeds was tested. Some representative experimental results are shown in Figure 6c. It can be seen that there is no measurement error when the speed is less than 100 rpm. As the speed increases, the measurement error gradually increases, and the maximum error is less than 2%. The reason is that when the speed is too fast, the friction contact between the stator and rotor is insufficient, and there may be no frictional electrical signal output, thereby introducing errors. Therefore, the angle measurement error of the sensor in the range of 0 to 1000 rpm is defined as 2%.

### 3.5. Power Generation Characteristics Experiment Results

**Figure 7:**
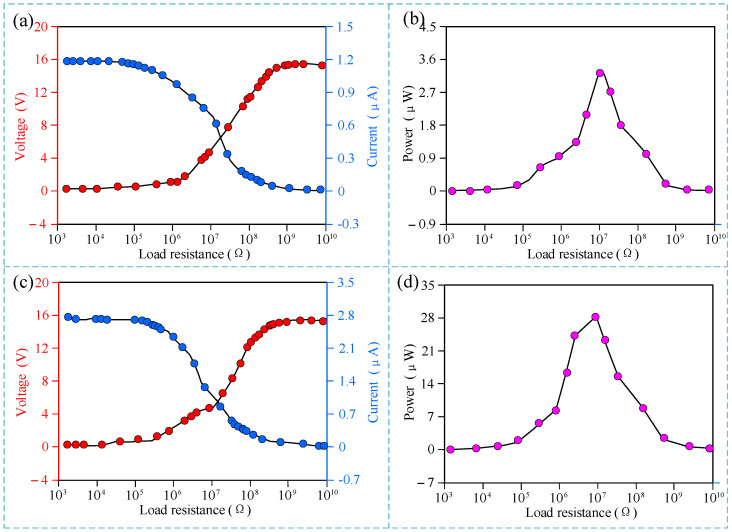
Experimental results of the power generation characteristics. (**a**) Output voltage and current of a single-layer electrode under different loads; (**b**) output power of a single-layer electrode under different loads; (**c**) output voltage and current of three-layer electrodes connected in parallel under different loads; (**d**) output power of three-layer electrodes connected in parallel under different loads.

The experimental results of the sensor’s power generation characteristics are shown in Figure 7. As shown in Figure 7a, with the increase in external load, the output voltage of a single electrode of the sensor gradually increases while the output current decreases, and the maximum values of voltage and current are 15.9 V and 1.17 μA, respectively, which is consistent with Ohm’s law. The output power of the sensor’s single electrode under different external loads was further tested. As shown in the experimental results in Figure 7b, with the increase in external load, the output power of the sensor shows a trend of first increasing and then decreasing, and the generated output power reaches a maximum value of 3.5 μW when the external load is 10 MΩ.

Since the sensor contains three electrodes with the same structure, the power generation characteristics of the three electrodes connected in parallel were further tested. The results are shown in Figure 7c,d. As shown in Figure 7c, with the increase in external load, the output voltage and current of the three electrodes are consistent with those of a single electrode. However, the voltage value is basically consistent with that of a single electrode, while the current value is about 2.4 times that of a single electrode. The reason is that since the three electrodes are connected in parallel, the voltage value after parallel connection remains unchanged, while the current value is the superposition of the current values of multiple electrodes. Therefore, the voltage value remains unchanged, while the current value should theoretically be three times that of a single electrode. However, when the three electrodes are used simultaneously, due to processing accuracy, there are slight differences in the friction contact degree of the three electrodes, so the multiple of the parallel connection is less than three times. Similarly, the output power curve of the three electrodes connected in parallel is also the same as that of a single electrode, and the generated output power reaches a maximum value of 29.1 μW when the external load is 10 MΩ. This power value is about 8.3 times that of a single electrode.

### 3.6. Environmental Adaptability Experiment Results

**Figure 8:**
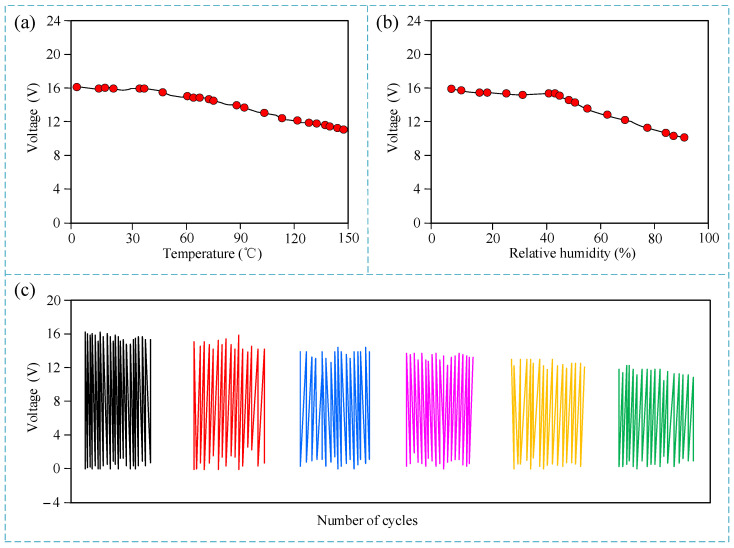
Experimental results of environmental adaptability. (**a**) Output voltage under different working temperatures; (**b**) output voltage under different relative humidity; (**c**) output voltage waveform under different cycles.

The sensor operates in an underground environment with varying temperature and humidity. Therefore, the output characteristics of the sensor under different temperatures and humidities were tested. As shown in Figure 8a,b, the experimental results show that within the temperature range of 0 to 150 °C and the relative humidity range of 0 to 90%, the output voltage of the sensor gradually decreases with the increase in temperature and relative humidity. The voltage amplitudes after the decrease are 11.3 V and 10.7 V, respectively. The output voltage pulse signal of the sensor is read by a subsequent microprocessor. The microprocessor is a digital chip that relies on its internal pulse interrupt port to read the voltage level state of the sensor input. At this time, as long as the input voltage amplitude is greater than 2 V, it can be determined as a high level by the microprocessor. Under the operating environment with temperature and humidity changes, the voltage amplitude after the decrease is still much greater than 2 V, which has no effect on the sensor. Therefore, the operating temperature and humidity ranges of the sensor are defined as 0 to 150 °C and 0 to 90%, respectively. The output characteristics of the sensor under different operating cycles were further tested. It can be seen that with the increase in operating cycles, the output voltage amplitude of the sensor gradually decreases, and when the sensor operates to 2 × 10^5^ times, the output voltage is about 13.7 V, which is still much greater than the recognition standard of high level. This proves the stability of the sensor during long-term operation.

## 4. Discussion

Despite the promising results, the current power output remains a limitation, as it is only sufficient to sustain the sensor itself and does not provide enough power to support other downhole measurement tools. Enhancing the sensor’s power output is a critical area for future research, which will focus on innovative structural designs and the use of high-power density nanomaterials. These improvements could potentially increase drilling efficiency and reduce operational costs, addressing a key challenge in the development of downhole sensors. Continued efforts in this direction are expected to further advance the field of drilling technology and measurement capabilities.

## 5. Conclusions

This study successfully developed a multifunctional downhole drilling motor rotational speed sensor utilizing triboelectric nanogenerator technology. The sensor operates reliably across a temperature range of 0 to 150 °C and a humidity range of 0 to 90%. It measures rotational speed with an accuracy of less than 2.5% error within a range of 0 to 1000 rpm, and angular displacement with a resolution of 60 degrees and an error of less than 2% over 360 degrees. Additionally, it determines rotational direction and features self-powered functionality, generating a maximum power output of 29.1 μW with a 10 MΩ load. These advancements differentiate the sensor from conventional downhole sensors, significantly improving downhole data collection by integrating multiple measurements and offering a more comprehensive analysis of drilling conditions. Moreover, the sensor’s self-powered capability reduces operational inefficiencies caused by frequent surface trips for battery replacements.

## Data Availability

All data and models generated or used during the study appear in the submitted article.
